# Low oral health literacy, dental caries, and school features are associated with reasons for seeking dental services among adolescents

**DOI:** 10.1590/1980-549720240066

**Published:** 2024-12-09

**Authors:** Roanny Torres Lopes, Érick Tássio Barbosa Neves, Laio da Costa Dutra, Ramon Targino Firmino, Larissa Chaves Morais de Lima, Fernanda de Morais Ferreira, Ana Flávia Granville-Garcia

**Affiliations:** I Universidade Estadual da Paraíba – Campina Grande (PB), Brazil.; II Universidade Estadual da Paraíba – Araruna (PB), Brazil.; III Faculdade São Francisco – Cajazeiras (PB), Brazil.; IV Universidade Federal de Campina Grande – Patos (PB), Brazil.; V Universidade Federal de Minas Gerais – Belo Horizonte (MG), Brazil.

**Keywords:** Oral health, Schools, Adolescent, Dental health service, Dental caries, Health literacy, Saúde bucal, Escolas, Adolescente, Serviço de saúde bucal, Cárie dentária, Alfabetização em saúde

## Abstract

**Objective:**

The present study aimed to investigate associations between reasons for seeking dental services, considering the last dental appointment of adolescents, and their educational features, socioeconomic and oral health status, and oral health literacy.

**Methods:**

A cross-sectional study was conducted with 746 adolescents aged 15–19 years in Campina Grande (Paraíba), Brazil. Parents/guardians answered a socioeconomic questionnaire. Adolescents answered a questionnaire on oral health and the Brazilian Rapid Estimate of Adult Literacy in Dentistry. Features of the school environment were investigated through cluster analysis, using type of school and school grade retention. Adolescents were clinically examined for dental caries diagnosis by two dentists (κ>0.80), using the Nyvad criteria, in school facilities. Data were submitted to robust multilevel logistic regression for complex samples (α=5%).

**Results:**

At the individual level, low maternal schooling (odds ratio [OR] 1.06; 95%CI 1.01–1.10), low oral health literacy (OR 0.99; 95%CI 0.98–0.99), and dental caries (OR 1.09; 95%CI 1.01-1.18) remained associated with the reasons for the last dental appointment. The school environment was also associated with the outcome (OR 1.07; 95%CI 1.00–1.15).

**Conclusion:**

Maternal schooling of less than eight years of study, low oral health literacy, cavitated caries, and unfavorable school environment were associated with seeking dental treatment.

## RESUMO

**Objetivo:**

O presente estudo teve como objetivo investigar associações entre os motivos da busca por serviços odontológicos, considerando a última consulta odontológica de adolescentes, e suas características escolares, status socioeconômico e bucal e alfabetização em saúde bucal.

**Métodos:**

Foi realizado um estudo transversal com 746 adolescentes de 15–19 anos em Campina Grande (Paraíba), Brasil. Os pais/responsáveis responderam a um questionário socioeconômico. Os adolescentes responderam a um questionário de saúde bucal e à versão brasileira do *Rapid Estimate of Adult Literacy in Dentistry* (rápida estimativa de alfabetização de adultos em odontologia). As características do contexto escolar foram investigadas por meio de análise de *cluster*, utilizando tipo de escola e taxa de retenção escolar. Os adolescentes foram examinados clinicamente para diagnóstico de cárie dentária por dois dentistas (κ>0,80), usando os critérios de Nyvad, nas instalações das escolas. Os dados foram submetidos à regressão logística multinível robusta para amostras complexas (α=5%).

**Resultados:**

No nível individual, a baixa escolaridade da mãe (razão de probabilidade [OR] 1,06; IC95% 1,01–1,10), alfabetização em saúde bucal inferior (OR 0,99; IC95% 0,98–0,99) e cárie dentária (OR 1,09; IC95% 1,01–1,18) permaneceram associados ao motivo da última consulta odontológica. O ambiente escolar também foi associado ao desfecho (OR 1,07; IC95% 1,00–1,15).

**Conclusão:**

Escolaridade materna com menos de oito anos de estudo, baixa literacia em saúde bucal, cárie cavitada e ambiente escolar desfavorável foram associados à procura por tratamento odontológico.

## INTRODUCTION

Adolescence is a phase of life marked by individual changes and social, economic, environmental, and behavioral factors, as well as regional differences that can influence the search for dental services and the prioritization of preventive care^1^. Factors at the individual level, such as dental anxiety, difficult access to dental services, and inadequate communication between patient and dentist can affect the search for dental treatment in this age group^2^. Thus, adolescence is an important phase to be studied, especially with regard to the search for dental services, as a transition occurs in this period, with the responsibility of health care switching from parents/guardians to adolescents themselves^3^.

In Brazil, there are public health programs aimed at promoting equal access to health services. In 2004, the National Oral Health Policy – Smiling Brazil (*Política Nacional de Saúde Bucal – Brasil Sorridente*) was launched, which expanded access to free dental care in primary care and at specialized level, offering actions for health promotion, prevention, and recovery^4,5^. However, it is observed that among adolescents aged 15–19 years, 65.1% reported need for dental treatment and 13.6% had never visited the dentist^5^. Studies have reported that the main reasons for seeking dental treatment in adolescence and other age groups are symptoms and established problems, such as pain, dental caries, or periodontal disorders^6,7^. Socioeconomic deprivation may also be associated with the search for dental services, with a higher frequency of curative care over preventive care^8,9^.

Oral health literacy (OHL) has been investigated in recent years as one of the factors associated with oral health practices^10,11^. OHL is a social and structural health determinant that suggests the capacity to understand and use oral health information^12^. A recent study found an association between low OHL and the search for dental care in cases of pain in adults^13^. However, the association between OHL and reasons for the last dental appointment among adolescents had not been previously investigated. Studies have reported that low OHL level contributes to missed appointments^14^, whereas high OHL relates to regular checkups^15^ and better patient-dentist communication^16^. Therefore, OHL may be associated to reasons for the last dental appointment, as a higher level of understanding regarding oral health favors the adoption of preventive attitudes^15^.

The school environment is another factor that should be considered. In this setting, adolescents develop cognitive and behavioral skills, expand their social relations, and establish goals for the future^17^. Studies have reported that the school environment contributes to the establishment of preventive oral health behaviors^6,18^.

The objective of this study was to investigate whether there is association between sociodemographic factors, clinical factors, OHL, and school environment with reasons for the last dental visit among adolescents.

## METHODS

### Study design

An analytical, representative, school-based, cross-sectional study was conducted with adolescents aged 15–19 years in the city of Campina Grande, state of Paraíba, Brazil. Data collection occurred from October 2016 to July 2017.

### Eligibility criteria

Adolescents in the target age group enrolled in public and private schools were included. Individuals with speech, hearing, cognitive, or behavioral problems that would impair the conduction of the study were excluded.

### Sample size calculation

The sample size was calculated for analytical studies comparing two independent proportions through G*Power software, version 3.1 (Franz Faul, Universitat Kiel, Germany), adopting a 95% significance level and 80% power. The proportion of adolescents in favorable and unfavorable educational environment that visited a dentist for the resolution of symptoms was 48.9 and 63.0%, respectively, based on the results of a pilot study, determining a minimum sample of 388 adolescents. The study population was obtained through two-stage cluster sampling (random selection of schools within each cluster, proportionally to the administrative district, followed by the selection of individuals using a simple random sampling procedure) to ensure greater homogeneity among individuals in each cluster. The correction factor of 1.6 was used in the present study^19,20^ leading to a sample of 621 participants. A 20% adjustment was made to compensate for possible dropouts, resulting in a desired sample of 777 adolescents.

### Pilot study

A pilot study was conducted with a total of 50 adolescents chosen by convenience who completed questionnaires and were submitted to clinical examinations. These adolescents were not included in the main study sample. The results of this pilot study revealed no need for changes to the proposed methods.

### Ethical considerations

This study was conducted in accordance with the ethical precepts stipulated in the Declaration of Helsinki and Resolution No. 466/2012 of the National Board of Health and received approval from the Human Research Ethics Committee of the *Universidade Estadual da Paraíba* (55953516.2.1001.5187).

### Variables

Parents/guardians completed a sociodemographic questionnaire informing adolescents’ sex (male and female), skin color (black, brown, white, and yellow) and maternal schooling (≤8 years of study or >8 years of study). Socioeconomic status was determined using the Brazilian Economic Classification Criteria (*Critério de Classificação Econômica Brasil*), which categorized families into classes A, B, C, D, and E based on the schooling of the household head, consumer goods, water supply, and residence on a paved street^21^. In the present study, classes A and B were considered indicative of higher socioeconomic status, whereas classes C, D, and E were considered indicative of lower socioeconomic status^22^.

The other questionnaires were completed by adolescents individually in a separate room at school and at times previously scheduled by the administration. Adolescents answered the oral health questionnaire addressing the type of dental service used with the following question "Where was your last dental appointment?", with the answer options "1-Public service or 2-Private service (including health plan or agreement)"; and the reason for seeking dental care "What was the reason for your last dental appointment?", with the following answers: 1-Revision, prevention, or check-up and 2-Pain/treatment."

OHL was investigated using the Brazilian version of the Rapid Estimate of Adult Literacy in Dentistry (BREALD-30), which has been validated for use in adolescents. This instrument measures functional OHL based on the capacity to recognize, read, or write words related to oral health. BREALD-30 is a screening tool composed of 30 dental terms. Adolescents were asked to read the words aloud. Words with no pronunciation or intonation errors were attributed one point and those with errors were scored zero. Higher scores indicate higher OHL levels^12^. The BREALD-30 instrument was administered by two interviewers, accompanied by four assistants. Agreement was calculated using Kappa (κ) statistics (word-by-word agreement) and intraclass correlation coefficient (ICC) scores (total scores agreement). In the present study, OHL was treated as a quantitative variable.

### School environment

The school environment was assessed using cluster analysis considering the type of school (public or private) and school grade retention (SGR) (students retained in the same grade after two or more failures)^22,23^. For statistical purposes, school environment was categorized as favorable (private school and lower SGR) or unfavorable (public school and higher SGR). The chi-square test was used to determine independence between groups and cluster validity. School data were obtained from the 2017 school census published by the National Institute of Educational Studies and Researches Anísio Teixeira (*Instituto Nacional de Estudos e Pesquisas Educacionais Anísio Teixeira;* INEP, 2017)^24^. The cluster of students in public schools with high SGR rate was considered unfavorable as the literature has demonstrated that dental problem prevalence is higher among students enrolled in public schools. Similarly, the repetition of consecutive grades can exert a negative influence on the oral health of adolescents^22^. The neighborhood income was determined based on data from the Brazilian Institute of Geography and Statistics (*Instituto Brasileiro de Geografia e Estatística*; IBGE, 2017)^25^.

### Clinical examination

Adolescents were positioned in front of the examiner, who dictated information for the assistant to record. The examination was performed using a headlamp (Petzl Zoom headlamp, Petzl America, Clearfield, UT, USA), mouth mirror, millimeter probe (OMS-621, Trinity^®^, Campo Mourão, PA, Brazil), and sterile gauze to dry the teeth. After examination, fluoride gel at 2% concentration was applied as a caries prevention measure for students with active white spots.

Dental cavities were assessed according to the Nyvad criteria, which are used for recognizing dental caries in the initial phases and their progression up to tooth loss, enabling greater accuracy in the diagnosis, whether for clinical or research purposes^26^. In this study, Nyvad codes 3 (active cavitated lesion) and 6 (inactive cavitated lesion) were considered on at least one anterior or posterior tooth, as these codes represent the worst clinical presentation of the disease.

### Training and calibration exercises

Researchers were trained by an experienced dentist who served as the "gold standard" for the administration of the BREALD-30 instrument. The first step consisted of a theoretical discussion on pronunciation criteria: adequate reading of words, repetition or substitution of terms, change in the stressed syllable, and paused reading or with additional sounds. The following step was the assessment of videos of individuals with different OHL levels. Additional videos were used for the OHL assessment and the process was repeated after seven days^12,27^ to enable the calculation of inter-examiner (κ 0.87–0.88) and intra-examiner (κ 0.87–0.89) agreement. The ICC was used to determine agreement regarding the OHL scores: ICC=0.987 (95%CI 0.970–0.995) and 0.874 (95%CI 0.860–0.895) for inter-examiner agreement; ICC=0.973 (95%CI 0.921–0.991) and 0.994 (95%CI 0.982–0.998) for intra-examiner agreement.

The method proposed by Peres et al.^28^ was used to train and calibrate examiners for the Nyvad criteria application. Theoretical discussions were conducted, followed by the analysis of images, to which examiners applied the criteria to determine the diagnosis. Subsequently, adolescents were examined at an interval of seven days. An experienced dentist with expertise in dental caries diagnosis supervised all steps. Inter-examiner (κ 0.89–0.90) and intra-examiner (κ 0.88–0.90) agreement was considered satisfactory.

### Statistical analysis

Statistical analysis was conducted with the Statistical Package for Social Sciences (SPSS) version 22.0 (IBM Corp., Armonk, NY, USA), adopting a 95% confidence level. Descriptive statistics were performed with the calculation of absolute and relative frequencies for the sample characterization. Multilevel logistic regression for complex samples was used to determine associations between independent variables and the reason for the last dental appointment (outcome). The odds ratio (OR) was the effect measure used to indicate the probability of an event occurring in each group.

The conditional variance of the outcome exceeded the conditional mean (overdispersion); for this reason, adjusted and unadjusted robust multilevel log-linear negative binomial regressions were performed^29^, considering the complex sampling design to evaluate the association between individual/contextual variables and the reason for seeking dental services.

Prior to the multilevel analysis, school features were grouped using cluster analysis to investigate the school environment. This analysis indicated the formation of two clusters: private schools with lower SGR and public schools with higher SGR. The aforementioned variables were selected for the cluster analysis because these predictors can indicate school's social aspects and the quality of the learning environment. The optimal number of clusters found was two groups and the general profile of the first cluster was mainly composed of students from public schools with higher SGR rate. The second cluster was predominantly composed of adolescents from private schools with lower SGR rate. Differences between groups were investigated using the chi-square test. The cluster analysis validity was confirmed.

For interpretation purposes, the first cluster was named as unfavorable school environment, while the second cluster was considered a favorable school environment. The variable formed in the cluster analysis was used as a predictor in the statistical model. A two-stage (adolescents and schools) multilevel analysis was conducted considering individual and contextual factors associated with the reason for seeking dental services.

In the multilevel analysis, a null model was used to examine the effects of variables and enable a better fit. Model 1 only included variables at the individual level. Those with p-value (p)<0.200 and adjustment factors were incorporated into the second model. Contextual and individual variables were incorporated into the third model. Variables with p<0.050 were considered associated with the reason for the last dental appointment. The appropriate model fit was determined based on deviance values (-2 log likelihood).

## RESULTS

This study had a final sample of 746 adolescents. The 4% loss rate was due to absences after three attempts to conduct examinations. Female adolescents (59.5%), lower socioeconomic level (57.4%), and maternal schooling of more than eight years of study (59.7%) predominated in the sample. The most prevalent reason for the last dental appointment was treatment or the resolution of pain symptoms (70.1%). A total of 70.2% of adolescents studied in an unfavorable school environment (Table 1).

**Table 1:** Characterization of 746 adolescents aged 15–19 years who participated in the present study in 2017. Campina Grande (PB), Brazil.

Quantitative variable		Mean (standard deviation)	
Oral health literacy		20.35 (5.06)	
Categorical variables		Frequency	
		n	%
Sex			
	Male	302	40.5
	Female	444	59.5
Self-declared skin color			
	Non-white	535	71.7
	White	211	28.3
Socioeconomic status			
	Classes A and B	318	42.6
	Classes C, D and E	428	57.4
Maternal schooling (years of study)			
	<8	299	40.3
	≥8	443	59.7
Cavitated caries			
	Yes	310	41.6
	No	436	58.4
Type of dental service			
	Public	264	40.4
	Private	389	59.6
Reason for appointment			
	Prevention	194	29.9
	Treatment/symptoms	455	70.1
Contextual variable			
School environment			
	Unfavorable	497	70.2
	Favorable	211	29.8

In the unadjusted analysis, non-white skin color, socioeconomic status C/D/E, lower maternal schooling, lower OHL score, and unfavorable school environment were associated with seeking a dentist for treatment (Table 2).

**Table 2:** Unadjusted analysis of reasons for last dental appointment among the 746 study adolescents aged 15–19 years in 2017. Campina Grande (PB), Brazil.

Reason for appointment				Bivariate Unadjusted OR*	
Categorical variables		Prevention n (%)	Treatment n (%)	(95%CI)	p-value
Sex					
	Male	84 (43.3)	117 (38.9)	1.02 (0.98–1.07)	0.260
	Female	110 (56.7)	278 (61.1)	1.00	-
Self-declared skin color					
	Non-white	130 (67.0)	339 (74.5)	1.05 (0.99–1.10)	0.050
	White	64 (33.0)	116 (25.5)	1.00	-
Socioeconomic status					
	Classes A and B	105 (54.1)	180 (39.6)	1.00	-
	Classes C, D and E	89 (45.9)	275 (60.4)	1.08 (1.03–1.13)	<0.010
Maternal schooling (years of study)					
	<8	50 (26.2)	200 (44.1)	1.09 (1.05–1.14)	<0.010
	≥8	141 (73.8)	254 (55.9)	1.00	-
Cavitated caries					
	Yes	41 (21.1)	227 (49.9)	1.11 (0.97–1.27)	0.120
	No	153 (78.9)	228 (50.1)	1.00	-
Type of dental service					
	Public	69 (37.1)	176 (41.0)	1.00	-
	Private	117 (62.9)	253 (59.0)	0.97 (0.93–1.01)	0.230
Contextual variable					
School environment					
	Unfavorable	97 (52.2)	326 (75.6)	1.14 (1.07–1.20)	<0.010
	Favorable	89 (47.8)	105 (24.4)	1.00	-
Income of school neighborhood		-	-	1.00 (1.00–1.01)	0.079
	Quantitative variable	Mean (SD)	Mean (SD)		
	Oral health literacy	22.4 (4.33)	19.8 (4.89)	0.98 (0.98–0.99)	<0.010

OR: odds ratio; CI: confidence interval; SD: standard deviation.

* Variables incorporated into adjusted model.

Socioeconomic status and skin color lost statistical significance in the adjusted analysis. In the multilevel analysis, the null model was used to evaluate the effects of variables; in model 2, individual variables were included, showing the association between lower maternal schooling, lower OHL score, and cavitated caries and the reason for the last dental appointment for pain/treatment. In model 3, which also included contextual variables, the following variables remained associated with the last dental visit for reasons of symptoms/treatment: lower maternal schooling (OR 1.06; 95%CI 1.01–1.10), lower OHL score (OR 0.99; 95%CI 0.98–0.99), cavitated caries lesion (OR 1.09; 95%CI 1.01–1.18), and the contextual variable unfavorable school environment (OR 1.07; 95%CI 1.00–1.15) (Table 3).

**Table 3:** Adjusted analysis of reasons for last dental appointment among the 746 study adolescents aged 15–19 years in 2017. Campina Grande (PB), Brazil.

		Model 1 ("null") OR (95%CI)	p-value	Model 2 OR (95%CI)	p-value	Model 3 OR (95%CI)
Intercept		1.67 (1.62–1.73)		1.81 (1.72–1.91)		1.71 (1.51–1.94)
Individual variables						
Sex						
	Male	-	-	-	-	-
	Female	-	-	-	-	-
Self-declared skin color						
	Non-white	-	-	-	-	-
	White	-	-	-	-	-
Socioeconomic status						
	Classes A/B	-	-	-	-	-
	Classes C/D/E	-	-	-	-	-
Maternal schooling (years)						
	<8	-	<0.001	1.06 (1.03–1.08)	<0.001	1.06 (1.01–1.10)
	≥8	-	-	-	-	-
BREALD-30 score		-	<0.001	0.99 (0.99–0.99)	0.005	0.99 (0.98–0.99)
Cavitated caries						
	Yes		<0.001	1.10 (1.07–1.12)	0.020	1.09 (1.01–1.18)
	No	-	-	-	-	-
Type of dental service						
	Public	-	-	-	-	-
	Private	-	-	-	-	-
Contextual variables						
School environment						
	Unfavorable	-	-	-	0.037	1.07 (1.00–1.15)
	Favorable	-	-	-	-	-
Income of school neighborhood		-	-	-	-	-
Deviance (-2log likelihood)		2,450.03		2,210.12		2,205.09

Model 1: null; Model 2: individual variables were included (sex, self-declared skin color, social class, type of dental service, maternal schooling, breald-30 score, cavitated caries); Model 3: individual and contextual variables were included (maternal schooling, breald-30 score, cavitated caries, school neighborhood income and school environment). OR: odds ratio; CI: confidence interval; BREALD-30: Brazilian version of the Rapid Estimate of Adult Literacy in Dentistry.

## DISCUSSION

This study sought to understand whether, in addition to clinical factors, the school environment and OHL are associated with the reason for the last dental appointment. OHL, unfavorable school environment, dental caries, and maternal schooling of less than eight years of study were associated with the search for dental care for pain/treatment among adolescents.

The prevalence of seeking a dentist for treatment or pain relief was 70.1%. In the literature, the prevalence ranges from 39.1 to 61.1%. It is believed that the social context acts as a determining factor in relation to access to dental services, possibly due to differences in educational level, oral health self-assessment, and socioeconomic conditions that impact access to better quality health services^1,7,30^. Low health literacy is associated with difficulty seeking preventive dentistry and poor oral health conditions in adolescents^31^. Difficulty in understanding can contribute to low prioritization of dental services in this population and the search for care when oral diseases already present symptoms, which has a negative impact on oral health-related quality of life^7,32^. This situation has implications for the individual's health, and also impacts health services, which may have greater demand for emergency care.

From the sociodemographic aspects, maternal schooling was associated with the reason for the last dental appointment among adolescents. A previous study demonstrated that the prevalence of dental caries is higher among children whose mothers have low schooling^33^. It is possible that maternal schooling is associated with the search for dental services among adolescents, as parental choices still exert an impact on the decisions of individuals in this period. Thus, inadequate oral health perceptions of children or insufficient appreciation of oral health may increase the occurrence of seeking a dentist only to resolve symptoms^34,35^.

In this study, the odds of adolescents with cavitated lesions seeking dental services due to pain were 9% higher than those without cavitated lesions. This may be explained by the fact that toothache is a common consequence of advanced dental caries stages and is the main reason for adolescents to seek dental services, which makes dental caries a predictor of seeking care^32,36,37^. Adolescents who attend regular dental appointments are less likely to have dental caries^3^. Awareness campaigns and oral health prioritization could facilitate the incorporation of positive attitudes.

OHL is an important mediator of oral health behaviors. Adolescents with higher OHL scores had their last dental appointment mainly for prevention reasons. On the other hand, low OHL was associated with a low frequency of visits to the dentist among adolescents and the occurrence of dental problems, such as dental caries^38,39^. Higher OHL level may sensitize adolescents regarding the importance of preventive care and regular dental appointments, contributing to better oral health. Intervention strategies should be adopted to strengthen this indicator, which would enhance the effective communication between patients and dentists^40^.

In addition to raising awareness and prioritizing oral health among adolescents, it is necessary to develop a comprehensive public health policy that extends from the family nucleus to the health service, covering the school. The dentist is the most qualified professional to guide oral health promotion and prevention strategies and must act as an intermediary agent. The scope of services must be increasingly expanded to cover all regions and population groups, as socioeconomic disparities are still a limiting factor in the access for dental services. Furthermore, the context in which adolescents are inserted must be considered and the awareness about the importance of early oral care must be directed at their families, with the participation of the school, so that strategies are proposed to enhance positive attitudes that minimize the risks of developing oral diseases in adolescence.

Adolescents in situations of unfavorable school environment (public schools with higher SGR) were more likely to seek dental services for treatment and resolution of symptoms. Previous studies have reported that an unfavorable school environment contributes to inadequate oral health practices and is associated with dental caries^22,33^.

Schools are influential environments for the dissemination of practices and information that favor adolescents’ autonomy. Students enrolled in public schools in Brazil tend to perform worse academically than those enrolled in private schools. This situation is a reflection of the better infrastructure of private schools and the family's socioeconomic standard^41^, which consequently influences adolescents’ choices and health behaviors. A study demonstrated poorer oral health behaviors and greater exposure to health risks among students enrolled in public schools^18^, which corroborates the association with socioeconomic deprivation and a context of insufficient information related to the prevention of oral problems. Similarly, SGR may indicate low capacity to process information in general, including information related to dental care. A study conducted with 12-year-old adolescents demonstrated a twofold higher average number of dental caries in adolescents enrolled in public schools with higher SGR ^[22]^. These results underscore the need for health administrators and providers to prioritize schools as an environment for transformation, and social vulnerability must be considered and serve as a subsidy for public health policies.

Similar studies have observed medium to high OHL levels^22,42,43^. This can be considered a health indicator for these populations. Such studies reinforce that this variable is associated with individual clinical and behavioral health factors such as eating habits, oral hygiene, visits to the dentist, and toothache and dental caries experience. Lower OHL scores suggest worse functional OHL, which is associated with worse oral health behaviors. Thus, this study also contributes to providing information on OHL and emphasizes the importance of creating a national policy to strengthen it. Measures should be taken to ensure that this is implemented since childhood; however, Brazil faces basic difficulties in the quality of formal education, which is reflected in the perception and choices of individuals throughout life. There must be a joint effort among professionals and specialists, the health system, legislators, schools, and the general population to find ways to strengthen functional literacy and health.

This study has limitations. Since it is a cross-sectional study, cause and effect relationships cannot be established. In population-based studies, respondents’ reporting personal information may be biased. However, measures were adopted to ensure internal and external data reliability and the sample representativeness, such as calibration of researchers, sample calculation, pilot study, and multilevel analysis. Adolescents were also instructed on the confidentiality of information provided and on the importance of truthfully responding the questionnaire.

It can be concluded that maternal schooling of less than eight years of study, lower OHL, dental caries, and an unfavorable school environment were associated with the search for dental care for pain/treatment among adolescents aged 15–19 years.
